# Anthropogenic disruption of the night sky darkness in urban and rural areas

**DOI:** 10.1098/rsos.160541

**Published:** 2016-10-19

**Authors:** Salvador Bará

**Affiliations:** Applied Physics Department, Universidade de Santiago de Compostela, 15782 Santiago de Compostela, Galicia, Spain

**Keywords:** environmental monitoring, wide-area sensing, light pollution, atmospheric effects, photometry

## Abstract

The growing emissions of artificial light to the atmosphere are producing, among other effects, a significant increase of the night sky brightness (NSB) above its expected natural values. A permanent sensor network has been deployed in Galicia (northwest of Iberian peninsula) to monitor the anthropogenic disruption of the night sky darkness in a countrywide area. The network is composed of 14 detectors integrated in automated weather stations of MeteoGalicia, the Galician public meteorological agency. Zenithal NSB readings are taken every minute and the results are openly available in real time for researchers, interested stakeholders and the public at large through a dedicated website. The measurements allow one to assess the extent of the loss of the natural night in urban, periurban, transition and dark rural sites, as well as its daily and monthly time courses. Two metrics are introduced here to characterize the disruption of the night darkness across the year: the *significant magnitude* (*m*_1/3_) and the *moonlight modulation factor* (*γ*). The significant magnitude shows that in clear and moonless nights the zenithal night sky in the analysed urban settings is typically 14–23 times brighter than expected from a nominal natural dark sky. This factor lies in the range 7–8 in periurban sites, 1.6–2.5 in transition regions and 0.8–1.6 in rural and mountain dark sky places. The presence of clouds in urban areas strongly enhances the amount of scattered light, easily reaching amplification factors in excess of 25, in comparison with the light scattered in the same places under clear sky conditions. The periodic NSB modulation due to the Moon, still clearly visible in transition and rural places, is barely notable at periurban locations and is practically lost at urban sites.

## 1. Introduction

Life on Earth evolved across the geologic time scales under relatively stable cycles of light and darkness. The overall amount of optical radiation on the surface of our planet oscillates periodically, following the daily cycle associated with the rotation of the Earth, the monthly cycle associated with the phases of the Moon and the yearly cycle of the seasons that modifies the relative lengths of the days and the nights throughout the year. These periodic patterns of light and darkness, modulated by local meteorological conditions, determine the time course of the radiant energy available to drive fundamental vital processes of many species. Furthermore, light is a key carrier of information about the opportunities and threats posed by the environment. It is therefore not surprising that a wide range of adaptations have been developed across evolutionary history, enabling organisms to anticipate periodic illumination changes and make the most efficient use of them [1,2]. A relevant number of species are nocturnal or need the darkness of the night for survival. The night is a realm full of life.

The natural darkness is nowadays at risk in large regions of the world, due to the spread of poorly designed outdoor lighting systems. Photons spilled from often overpowered and inadequately shielded streetlights travel long distances across the atmosphere impinging—either directly or after undergoing one or multiple scattering events—on places that were not intended to be lighted. The result is a perceptible increase of the amount of optical radiation present at night-time in areas that would otherwise be dark [3–5]. Artificial light at night has been shown to be a powerful disruptor of biological processes at the individual, species, community and, potentially, whole ecosystem scales [1,6–10]. The environmental effects of artificial light at night, however, are not restricted to the direct biological affectations. As of 2005, lighting consumed 19% of the electric energy produced worldwide [11] and indirectly contributed a relevant share of greenhouse gas emissions. There is also some evidence of the role of outdoor lighting in the nocturnal dynamics of chemical pollutants over cities [12]. The growing level of artificial skyglow also has detrimental consequences for science and culture: it is progressively hindering the ability to carry out accurate measurements in formerly first-class astronomical observatories located now too close to highly populated areas, and contributes to the loss of relevant aspects of the intangible heritage of humanity, in particular those associated with the contemplation of the starry sky. Overall, the anthropogenic disruption of the natural night sky darkness is a complex and multidimensional phenomenon that deserves an interdisciplinary approach [13–15].

Monitoring the artificial light emissions and measuring their effects at global and local scales is a necessary step for a better understanding of this phenomenon, as well as a prerequisite for making informed decisions on public policies concerning outdoor lighting systems.

Artificial light emissions can be directly monitored at a global scale by means of spaceborne radiometers like the Visible Infrared Imaging Radiometer Suite—Day-Night Band (VIIRS-DNB) of NASA's Suomi-NPP satellite, in orbit around the Earth at an altitude of 824 km with inclination 98.2°. VIIRS-DNB products provide panchromatic (500–900 nm), 14-bit depth radiance data of terrestrial sources with a low light imaging detection limit of 2 × 10^–11^ W cm^−2^ sr^−1^ and a ground footprint of 742 × 742 m. Although not free from some limitations, VIIRS-DNB measurements represent a significant improvement in comparison with previously available datasets like the ones of the Defense Meteorological Satellite Program Operational Linescan System (DMSP-OLS) [16]. An additional source of information on Earth's night-time lights is the DSLR imagery acquired from the Crew Earth Observations (CEO) facility of the International Space Station (ISS) [17]. Albeit not radiometrically calibrated in origin, high-resolution ISS images provide some basis for determining the different kinds of light sources composing the overall city emissions, taking advantage of the trichromatic information contained in their RGB channels [18]. At smaller spatial scales, high-resolution data (1 m or less) can be gathered by means of airborne radiometers and hyperspectral imagers [19–21].

The emissions of artificial light into the atmosphere give rise to a wide range of effects, including the increase of the skyglow at sites located several tens, or even hundreds, of kilometres away from the sources. These effects are not determined by the light sources alone: the atmosphere plays a key role redistributing the emitted energy through several light propagation related processes, including the scattering and absorption of the optical radiation. Both light sources and meteorological conditions are equally important when it comes to forming the light field in the nocturnal environment.

A widely used indicator of the severity of the disruption of the natural night conditions at any given place is the increment of the zenithal night sky brightness (NSB) over its expected natural levels. It can be measured with dedicated radiometers, all-sky cameras or astronomical narrow-field CCD photometers operating in different photometric bands [22–24]. Besides being useful for characterizing the local night-time conditions, monitoring the zenithal NSB is an essential tool for calibrating and/or validating the predictions of competing quantitative models that aim to describe the propagation of the light pollution through the atmosphere [25–30]. Wide datasets of measurements of this parameter have been instrumental for the calibration of the new World Atlas of artificial NSB [4].

The zenithal NSB is a time-dependent magnitude that shows a high degree of variability, both within any given night and between consecutive nights, due to the daily dynamics of urban lighting and the changing atmospheric conditions, especially in what concerns the aerosol content distribution and the presence and types of clouds. The radiometric characteristics of the sources and the state of the atmosphere determine the amount of light scattered in the direction of the observer by the air column located above. The overall sky brightness also includes contributions from natural sources, like atmospheric skyglow, zodiacal light and light from the Moon and other celestial bodies. Given the intrinsic variability of this parameter, any relevant description of the light pollution status of a given site requires the continuous monitoring of the zenithal NSB, with enough time resolution to keep track of the changes, and during extended periods of time in order to establish suitable baselines against which to compare potential long-term tendencies.

We describe in this paper the structure, deployment and science results of the Galician NSB Monitoring Network, a countrywide permanent detector network integrated in the automated weather stations of MeteoGalicia, the Galician public meteorology agency [31]. This network, fully operational since 2014, provides real-time data on the zenithal NSB at 14 selected locations distributed throughout the country, encompassing urban and periurban areas, transition regions and dark rural and mountain sites.

## 2. Material and methods

### 2.1. Night sky brightness: definition and units

NSB is a convenient shorthand term for the spectral radiance of the night sky, averaged within the field of view of the detector, and integrated across wavelengths after being weighted by the spectral function of a given photometric band.

Denoting the spectral radiance of the sky by *L*(ω; λ), the photometric band function by *T*(λ) and the function describing the field of view of the detector by *F*(ω), ω being the two-dimensional position vector of the points of the sky hemisphere above the observer (composed of their coordinates, e.g. azimuth and altitude over the horizon) and λ being the wavelength, the NSB *b* is given by

2.1 b=∬L(ω;λ) T(λ)F(ω) dΩ(ω) dλ,

where d*Ω*(ω) denotes an infinitesimal solid angle element around the direction ω. The angular integration is carried out within the 2*π* hemispheric field in front of the detector, and the spectral one is formally carried out for all wavelengths, although the effective dominion of integration is finite and bounded by the filter function. In writing equation (2.1), it has been assumed that the field of view function is normalized such that ∫F(ω) dΩ(ω)=1. SI units for the spectral radiance are W m^−2^ nm^−1^ sr^−1^. After wavelength integration, the overall radiance is given in W m^−2^ sr^−1^.

As deduced from this definition, the precise value of the NSB for a given location and time depends among other factors on the photometric band chosen to measure it. When this band matches the spectral sensitivity of the human visual system, as described by the CIE photopic spectral luminous efficiency function, *V_M_*(λ) [32], the resulting value, properly scaled by the maximum luminous efficacy factor 683 lm W^−1^, approaches the brightness perceived in foveal vision by the average human observer. Visual brightness is expressed in SI luminous units cd m^−2^. As 1 cd = 1 lm sr^−1^, and 1 lm is equal to (1/683) W of radiant power within the *V_M_*(λ) band, luminous brightness values can easily be transformed into equivalent radiances weighted by the *V_M_*(λ) filter.

A closely related photometric band, widely used in astrophysics, is the Johnson–Cousins V [33]. Its associated spectral function is a good approximation to *V_M_*(λ). A standard practice in this field is expressing the NSB in non-SI standard units *magnitudes per square arcsecond* (mag arcsec^−2^, or *mpsas*). The relationship between the *mpsas* in the V band (mag_V _arcsec^−2^, or m_V_) and the visual brightness *b* in nanoLamberts (nL) is given by equation (19) of [28] as

2.2 $$b[\mathrm{nL}]=34.08\exp(20.7233-0.92104\;m_{V}).$$

Taking into account that one Lambert equals 10^4^ *π*^−1^ cd m^−2^ and rewritting the exponentials in base 10 we get

2.3 $$b[\mathrm{cd}\cdot m^{-2}]=10.8\times 10^{4}\times 10^{\left(-0.4m_{V}\right)},$$

that, after division by the 683 lm W^−1^ efficacy factor, corresponds to the V-band weighted radiance

2.4 $$L_{V}[W\cdot m^{-2}\cdot {\mathrm{sr}}^{-1}]=158.1\times 10^{\left(-0.4m_{V}\right)}.$$

This is the radiance that would be reported by a detector fitted with a V-band filter with unit transmittance at the peak of the band, not to be confused with the total radiance of the incident beam in the visible range.

The brightness of a pristine dark sky, in clear and moonless nights and far from directions with conspicuous celestial sources (bright stars and planets of the Solar System, Milky Way and zodiacal light) still depends on the natural atmospheric airglow, whose intensity varies along the 11-year solar activity cycle, and on the residual contribution of the unresolved background stars. As a conventional baseline for assessing light pollution effects, the reference brightness of the natural dark sky is usually taken as 22.0 mag_V _arcsec^−2^ [4], equivalent to 0.17 mcd m^−2^, or to 0.25 µW m^−2^ sr^−1^ within the V band.

### 2.2. Stations network

**Figure 1.**
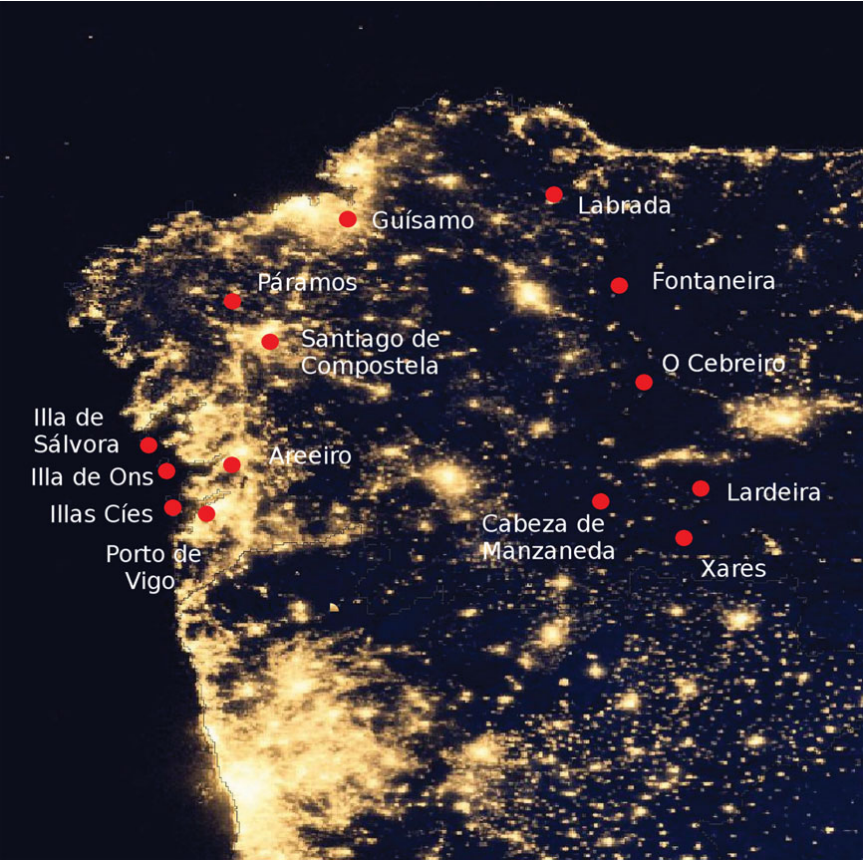
Geographical distribution of the detectors of the Galician Night Sky Brightness Monitoring Network, located at 14 MeteoGalicia weather stations. The brightest zone close to the lower border of the image is the metropolitan area of Porto (Portugal). Background night-time lights credit: NASA Earth Observatory (2012). Night Lights 2012—Flat map. Available online at http://earthobservatory.nasa.gov/NaturalHazards/view.php?id=79765, image by Robert Simmon, using Suomi NPP VIIRS data provided courtesy of Chris Elvidge (NOAA National Geophysical Data Center) (NASA Earth Observatory, 2012). Suomi NPP is the result of a partnership between NASA, NOAA, and the US Department of Defense.

After its first phase of deployment the Galician NSB Monitoring Network is composed of 14 detectors distributed across the territory of Galicia, autonomous community of Spain located north of Portugal in the northwest of the Iberian peninsula (figure 1). Galicia is located between 42° and 44° latitude north, and its oceanic climate is characterized by generally mild temperatures, winters with frequent episodes of overcast skies and strong rains and winds, driven by the low pressure systems coming from the Atlantic, and temperate summers. The average annual temperature is 13.3°C (averaging to 8.5° in winter and to 19° in summer) and the average rainfall is around 1000–1200 mm. The detectors are integrated in automated weather stations of MeteoGalicia, the Galician public meteorological and environmental information agency [34]. The measurement locations were selected to include a representative set of sites with different light pollution levels: urban centres with strong nocturnal emissions of artificial light, periurban places, transition regions like the Galician Atlantic Islands Maritime-Terrestrial National Park, and pristine dark areas in rural zones and the relatively unpopulated eastern mountains of the country. The station locations and types are listed in table 1.

**Table 1.** Location of the detectors and main parameters of the zenithal night sky brightness.

	Lon/Lat29TED-50				significant mag.			
area/station	UTMX	UTMY	altitude (m)	global mode	*m*_1/3_	*m*_1/10_	radiance ratio	moonlight factor γ
*urban centres*								
Santiago de Compostela	539222	4748551	305	19.2	19.1	19.3	14.5	0.30 × 10^−3^
Vigo (Harbour)	522594	4676863	7	18.7	18.6	18.8	22.9	0.23 × 10^−3^
*periurban sites*								
Guísamo	558515	4795645	176	19.8	19.8	20.0	7.6	0.42 × 10^−3^
Areeiro	527029	4694945	100	19.8	19.7	19.9	8.3	0.28 × 10^−3^
*transition regions*								
Illas Cíes	507678	4673514	25	20.9	21.3	21.7	1.9	1.46 × 10^−3^
Illa de Ons	505372	4692421	121	20.8	21.0	21.2	2.5	1.26 × 10^−3^
Illa de Sálvora	498997	4701610	24	21.2	21.5	21.9	1.6	2.81 × 10^−3^
Paramos	524636	4761290	369	21.1	21.1	21.2	2.3	1.20 × 10^−3^
*rural and mountain*								
Labrada	621411	4807150	662	21.3	21.5	21.6	1.6	1.90 × 10^−3^
Fontaneira	647041	4766600	917	21.4	21.7	22.1	1.3	1.75 × 10^−3^
O Cebreiro	660057	4730358	1310	21.5	21.6	21.7	1.4	1.72 × 10^−3^
Lardeira	682612	4694030	1620	21.5	21.8	22.2	1.2	1.53 × 10^−3^
Xares	674089	4675218	1762	21.5	22.3	23.1	0.8	2.41 × 10^−3^
Cabeza de Manzaneda	640477	4680282	1758	21.7	22.3	22.9	0.8	2.18 × 10^−3^

### 3.3. Detectors

The network is equipped with Sky Quality Meter (SQM-LR) detectors (Unihedron, Canada). They are based on a TSL237 high-sensitivity light-to-frequency converter (TAOS, USA) whose output is a square wave of frequency directly proportional to the irradiance incident on the photodiode. The nominal field of view of the detector has a Gaussian shape with angular full width 20° (FWHM), and the entrance optics includes an HOYA CM-500 IR blocking filter with non-zero spectral transmittance in the band 350–750 nm. A temperature sensor allows one to compensate the readings of the light sensor for thermal drifts in the range −40°C to +85°C. The detectors are enclosed in PVC protective housings with clear soda-lime glass windows, and linked through a serial RS232 connection to the meteorological station dataloggers.

The spectral band of the SQM devices closely matches the Johnson–Cousins V for wavelengths above 520 nm, showing increased relative sensitivity for shorter wavelengths. Owing to this fact, and depending on the spectra of the artificial light sources, SQM band measurements may slightly under or overestimate the corresponding V magnitude. For usual sources in realistic conditions, the differences typically lie below 0.3 mag arcsec^−2^ [35]. Henceforth, for the sake of accuracy, we will refer to the brightness values obtained in the SQM band as mag_SQM_ arcsec^−2^.

The overall uncertainty of the SQM measurements comes mainly from electrical, thermal and optical contributions, and according to Pravettoni *et al*. [36] is reported to be of order 0.186 mag_SQM_ arcsec^−2^ (standard overall uncertainty, including repeatability of the uncertainty measurement set-up). Intercomparisons of different detectors show the existence of systematic interdetector biases of about the same order of magnitude that can be easily compensated for in the data processing step.

### 2.4. Calibration and data acquisition

The 14 detectors installed in the network (plus six additional backup units) were intercalibrated at the instrument platform situated on the roof of the central building of MeteoGalicia headquarters, located within the city of Santiago de Compostela. Intercalibration zenithal brightness data were acquired at a rate of one measurement per minute during 40 consecutive nights in June–July 2013. A standard virtual detector was defined as the average of the actual ones (excluding outliers), and the regression curves relating the measurements of each physical detector to the standard one were subsequently determined. Linear regressions were sufficient in all cases to refer the individual detector readings to the common standard. During this calibration period, the transmittances of the glass windows of the housings were also measured. The window losses (mainly due to Fresnel reflections at the air–glass window interfaces) were found to be of order 0.1 mag_SQM_ arcsec^−2^, in agreement with the manufacturer specifications. This constant bias is subtracted from the raw measurements (the mag arcsec^−2^ is a negative logarithmic scale of brightness) in order to obtain the correct readings outside the windows.

In normal operation mode, the detectors continuously acquire one measurement of the zenithal sky brightness per minute. These raw measurements are transmitted in real time to the MeteoGalicia data servers and, after being corrected for the glass window losses, are stored and released for public distribution.

### 2.5. Public dissemination of results

The measurements gathered by the network are openly available in real time for researchers and the public at large in a dedicated section of the MeteoGalicia website [34]. The NSB plots of the last 48 h are shown for each station, as well as the average and maximum values of the NSB readings in mag_SQM _arcsec^−2^ for the last four nights. In all cases, the values displayed on the website correspond to 10 min averages of the native 1 min data, with the time expressed in UTC. Users can access through the same webpage to some relevant documents and related links, as well as to the whole set of meteorological variables measured at each station. There is an option for downloading user-defined NSB datasets, including any additional variable or group of variables of interest. The original 1 min NSB data, with complementary information about the intercalibration factors of the detectors, are freely available upon request.

## 3. Results

This section reports the main features of the statistical behaviour of the NSB at the different locations, based on the data gathered by the network during the year 2015. All stations were operational as of the beginning of this year, with the exception of the Illa de Sálvora one that began its data transmissions in the month of September. Most of the stations provided uninterrupted sets of data during the whole period considered. In a limited number of cases, there were some temporal data segments missing, due to communications and/or hardware failures produced by extreme weather conditions.

The time course of the zenithal sky brightness in any given night is determined by the hourly evolution of the artificial light emissions, the particular state of the atmosphere, and the contributions from natural light sources, both extraatmospheric (celestial bodies) and atmospheric (airglow and transient phenomena, like lightning). Examples of individual night plots of the zenithal sky brightness, also called scotograms, can be found in [37,38]. Here, we will focus on the statistical distribution of the NSB values in locations with different levels of light pollution, as well as on the progressive loss of the moonlight modulation signal as we approach places with strong light emissions.

### 3.1. Night sky brightness densitograms

**Figure 2.**
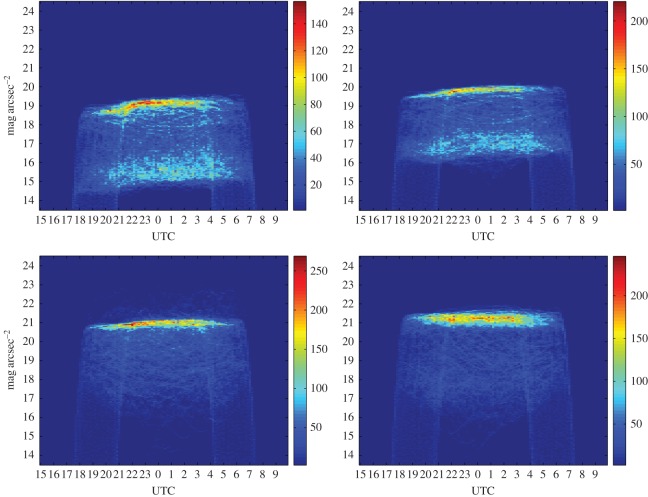
Densitograms of the zenithal night sky brightness at the stations of Santiago de Compostela: urban; Guísamo: periurban; Illas Cíes: transition; and Labrada: rural (from left to right and top to bottom). The colour scale indicates the absolute number of measurements of each brightness level at each time period of the night. The resolution is 0.05 mag_SQM_ arcsec^−2^ in brightness and 10 min in time (UTC).

Densitograms, also called density plots [37], are time-resolved histograms of the NSB. Figure 2 displays the densitograms corresponding to the stations of Santiago de Compostela (urban), Guísamo (periurban), Illas Cíes (transition) and Labrada (rural), computed using uninterrupted sets of measurements of the year 2015. The colour-bar scale gives the absolute number of records corresponding to each brightness level and time bin. The vertical axis spans the brightness values, with resolution of 0.05 mag_SQM_ arcsec^−2^. The horizontal axis corresponds to UTC time, with 10 min resolution. Civil time in Galicia is UTC + 1 in winter (CET), and UTC + 2 during the daylight savings period (CEST).

The densitograms provide a first insight on the behaviour of the zenithal skyglow. A typical densitogram contains several distinct features. The narrow zone of high concentration of measurements located in the region of high mag_SQM_ arcsec^−2^ values (darker skies) corresponds to the brightness recorded under moonless clear sky conditions. Inspection of the densitograms in figure 2 reveals that typical readings under these conditions range from 19 mag_SQM_ arcsec^−2^ at the urban centre of Santiago de Compostela (100 000 inhabitants) to more than 21 mag_SQM_ arcsec^−2^ at the rural Labrada site. Keep in mind that, due to the negative logarithmic definition of the magnitude scale, higher mag_SQM_ arcsec^−2^ values correspond to darker skies. It can also be seen that the zenithal darkness of the sky in urban and periurban areas (and, to a lesser extent, in the transition ones) increases steadly in the first hours of the night, due to the progressive switch-off of the ornamental and commercial lighting and the reduction of the road traffic flow. This effect is practically absent in dark rural sites like Labrada (bottom-right densitogram).

Another distinct feature of the densitograms of urban and periurban sites is the presence of a second region of concentration of measurements, located between 2.5 and 3.5 mag_SQM_ arcsec^−2^ below the previous one. Such difference in magnitudes corresponds to an increase of 10 to 25 times in brightness, and is mostly due to the multiplicative effects of the cloud cover [39]. Whereas in dark rural sites the presence of clouds tends to reduce the overall amount of radiation reaching the detectors (as they obscure the celestial light sources) and hence to increment the recorded mag_SQM_ arcsec^−2^ values, in highly illuminated places like urban and periurban areas the artificial light reflected on the base of the clouds significantly increases the zenithal sky brightness.

Typical densitograms also feature a set of nearly vertical lines, corresponding to the brightness values recorded during the evening and morning twilights, whose position along the temporal axis varies following the seasonal pattern of the year. The remaining set of lines distributed throughout the densitogram reflects the contributions of the Moon, which depend on its phase and zenithal distance, and comprise both the scattered moonlight and the direct radiance of the Moon when it falls within the field of view of the detector.

### 3.2. Night sky brightness histograms

**Figure 3.**
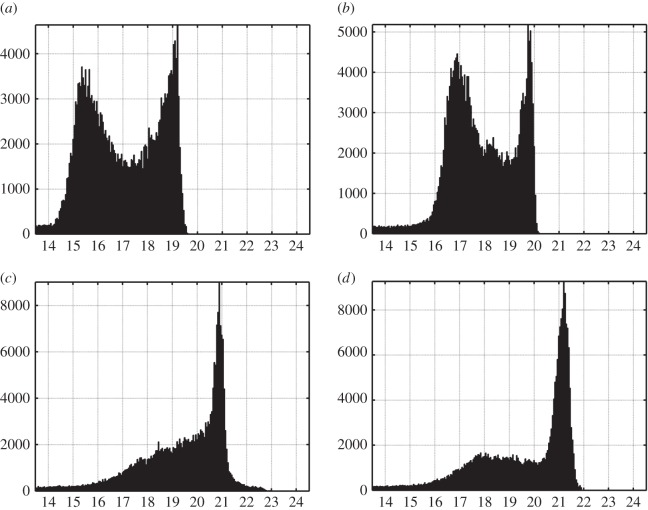
Histograms of the absolute frequencies of the zenithal night sky brightness recorded at the stations of (*a*) Santiago de Compostela, urban; (*b*) Guísamo, periurban; (*c*) Illas Cíes, transition; and (*d*) Labrada, rural. The horizontal axis corresponds to the brightness in mag_SQM_ arcsec^−2^ and the vertical one to the absolute number of measurements.

Absolute frequency histograms can be obtained by integrating the densitogram values across the temporal axis (figure 3). The histograms of urban and periurban sites are clearly bimodal, with the darkest peak (higher mag_SQM_ arcsec^−2^ value) corresponding to the most frequent readings obtained in moonless clear nights and the brightest one (lower mag_SQM_ arcsec^−2^ value) corresponding to the most frequent values obtained under overcast conditions. The absolute positions of the peaks reveal the prevalent sky brightness at these sites, and the distance between them along the horizontal axis gives the prevalent cloud amplification factor in mag_SQM_ arcsec^−2^ units. The cloud amplification factor, in natural units, indicates how many times the overcast sky is brighter than the same sky in clear nights. Recorded typical values of this factor in urban and periurban areas lie in the range from 10 (Guísamo) to 16 (Vigo), with an individual value close to 25 registered at the urban centre of Santiago de Compostela (100 000 inhabitants). Taking into account that the histogram modes for clear skies in Vigo and Santiago de Compostela are 21 and 13 times brighter, respectively, than the natural dark baseline (see global mode in table 1), this means that urban cloudy nights may be more than 300 times brighter than dark clear natural ones. The larger width of the brightest peak reflects the higher variability associated with the different cloud conditions (cloud structure, albedo and height above ground level). The width of the darkest peak, corresponding to clear nights, is mainly determined by the variability of the aerosol content in the atmosphere and the time course of roadway and traffic lighting.

In places with darker or nearly pristine skies, however, the bimodal structure of the histograms is much less pronounced or inexistent. The clouds in these places tend to darken the zenithal sky brightness below its natural values because there is little amount of artificial light to be reflected in the base of the clouds, and the cloud cover acts as a screen blocking the natural celestial light sources. The secondary peak visible in dark rural sites as Labrada (figure 3*d*) stems mainly from the scattered and direct moonlight contributions, clearly notable in the corresponding densitogram (figure 2, bottom right). The Illas Cíes site, close to some coastal towns, represents an intermediate situation.

Some extremely dark values (more than 22.0) can be recorded under particular circumstances, including thick cloud cover in dark areas or the temporary presence of obstacles over the detector window (e.g. thick snow layers in some winter nights). In the case of the Illas Cíes detector, there is another factor contributing to some unusual records, namely, the intermittent dense sea fog events experienced at the islands of the southern region of the Galician coast.

**Figure 4.**
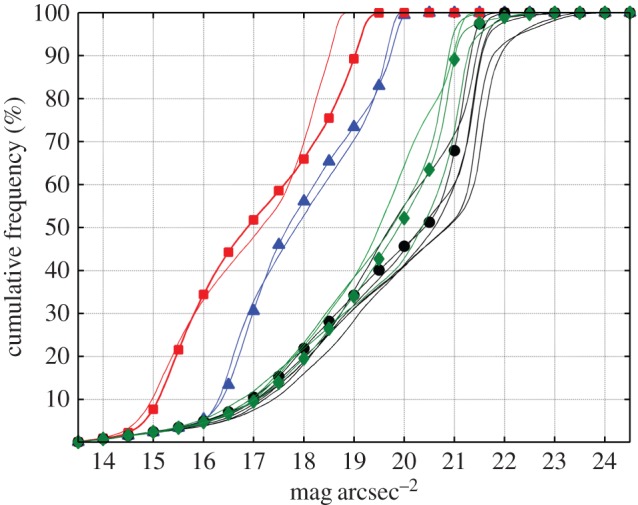
Cumulative frequencies of the zenithal night sky brightness (for mag_SQM_ arcsec^−2^> = 13.5) recorded at the 14 stations of the network. Red lines correspond to urban settings, blue to periurban, green to transition areas and black to rural dark sites. Symbols label particular stations: Santiago de Compostela (squares), Guísamo (triangles), Illas Cíes (diamonds) and Labrada (circles).

Figure 4 shows the relative cumulative histograms of the zenithal sky brightness values. According to the results shown in figure 3, the cumulative histograms in brightly illuminated urban and periurban sites saturate at smaller magnitude values, and the rising segment of their curves shows two distinct inflection points associated with the distribution of brightness driven by cloud reflections.

### 3.3. Significant magnitude

The high variability of the NSB makes it difficult to assign a single mag_SQM_ arcsec^−2^ value to characterize the light pollution level of any particular site. The NSB is an environmental variable that, like the wind speed or the ocean wave height, requires to be described by its statistical distribution. It is however useful for many applications to provide an index, even if only approximate, of the expected night brightness under typical conditions.

The global statistical mode of the distributions shown in figure 3 provides a first estimation of the typical darkness of a given site. In all stations of the network, the global mode for the year 2015 coincided with the darkest peak of the bimodal histograms, although this could not necessarily hold for urban and periurban sites if the presence of clouds was more frequent than the one recorded in the period under study. The values of the global mode are listed in the fifth column of table 1. This parameter, however, is sensitive to the choice of the size and centre of the brightness bins used to build the histograms.

**Figure 5.**
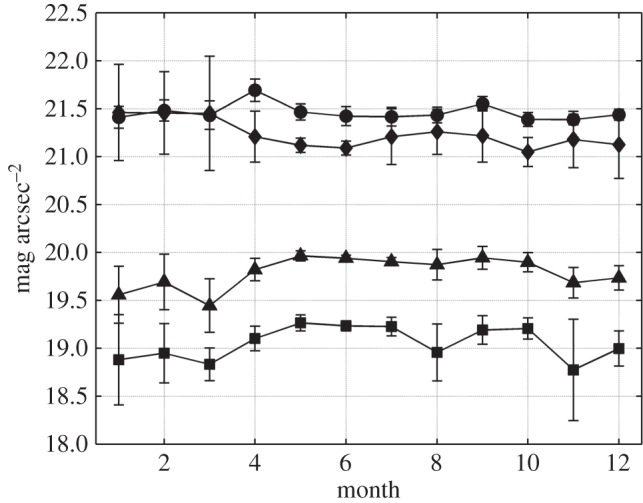
Time course of the monthly averaged significant magnitude *m*_1/3_ at the stations of Santiago de Compostela (squares), Guísamo (triangles), Illas Cíes (diamonds) and Labrada (circles). Error bars correspond to one standard deviation of the data.

As an alternative index, we define here the significant magnitude (*m*_1/3_) as the average of the highest third of NSB values recorded in conditions of astronomical darkness, with the Sun below −18° altitude (end of the astronomical twilight) and the Moon below −5° altitude. This index allows one to avoid most of the readings corresponding to light reflected by clouds, and provides a reasonable indication of the brightness expected in clear and moonless nights. The significant magnitude can be computed for any time period of interest. Figure 5 shows the evolution of the monthly values of *m*_1/3_ during the year 2015 for the four selected stations. The yearly values at each station are listed in the sixth column of table 1. The column ‘radiance ratio’ of this same table shows how many times an *m*_1/3_ sky is brighter than the reference dark natural sky. In urban centres, this ratio may be larger than 20, pointing to the severity of the light pollution effects in zones with high emissions of light. In transition and rural sites, typical values lie in the range 1.2–2.5. Values smaller than 1 are indicative of dark skies with frequent presence of clouds and, in some occassions, other potential obstacles in the detector field of view, like fallen snow.

A complementary index (*m*_1/10_) is the average of the upper tenth of values recorded in conditions of astronomical darkness. This index almost completely avoids the reflections in clouds in bright sites, at the price of giving more weight to some outlier dark values recorded at dark locations. The *m*_1/10_ values for the year 2015 at each station are also included in table 1. For urban and periurban locations, *m*_1/10_ is about 0.2 mag_SQM_ arcsec^−2^ (0.83 times) darker than *m*_1/3_. In transition and rural sites, the differences tend to be larger. The high values found in the mountain stations of Cabeza de Manzaneda and Xares reflect the good quality of their skies, although they are biased towards larger values due to the measurements made under thick cloud overcast conditions, and even under snow deposited on the detector during some of the coldest winter days.

### 3.4. The loss of the periodic moonlight signal

After the daily light cycle of day and night, the monthly cycle due to the Moon is the second main source of periodic variability of brightness in natural spaces. Recent research is unveiling its key role for a variety of biological processes, that not unexpectedly turn out to be significantly affected by the artificial emissions of light, especially in shoreline areas [40].

**Figure 6.**
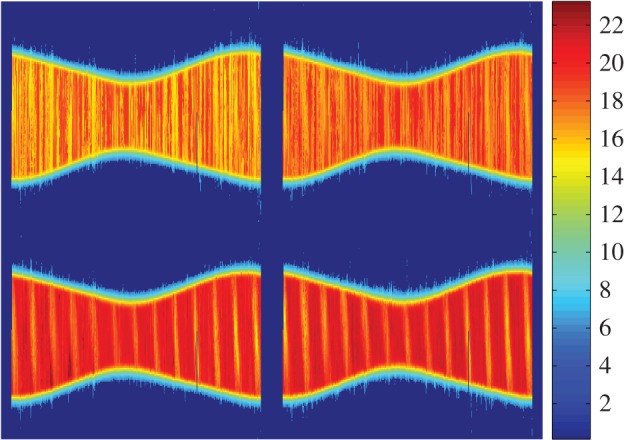
Daily evolution of the zenithal night sky brightness across the year at the stations (from left to right and top to bottom) of Santiago de Compostela, Guísamo, Illas Cíes and Labrada. The colour scale indicates the brightness in mag_SQM_ arcsec^−2^. Each column of the individual plots corresponds to a successive day, with the time increasing from top to bottom in 10 min intervals. The value displayed at each time interval is the median of 10 readings taken 1 min apart. The periodic moonlight signal is conspicuous in the transition (Illas Cíes) and rural (Labrada) sites but is practically lost at the urban (Santiago de Compostela) and periurban (Guísamo) ones.

The daily traces of the zenithal NSB can be displayed in the form of yearly plots as shown in figure 6. Each column of the plot corresponds to a correlative day of the year and the position within a column, starting from the top, correspods to the time of the day, in 10 min intervals. According to the usual practice in night studies, Julian days—beginning at noon of the corresponding civil day—are used in the horizontal axis. The top row of each graph thus corresponds to 12.00 h UTC and the middle row to midnight, that is, 00.00 UTC of the following civil day. The colour scale indicates the value (in mag_SQM_ arcsec^−2^) of the median of the 10 individual readings recorded at each time interval. The different length of the days and nights throughout the year is apparent.

A conspicuous feature of the graphs corresponding to transition and dark sites (figure 6, lower plots) is the presence of periodically spaced, slanted bands of alternating brighter and darker values. This periodic modulation of the NSB is due to the monthly cycle of the Moon. In periurban areas, however, the moonlight signal is hardly notable (figure 6, upper right), and in urban sites it is practically lost (figure 6, upper left).

**Figure 7.**
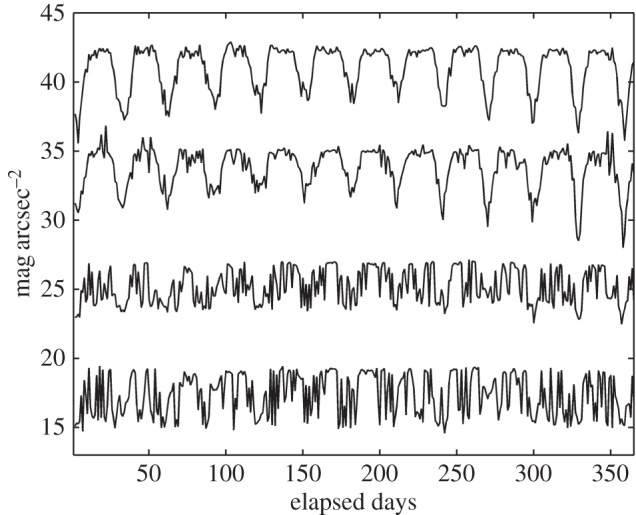
The yearly time course of the zenithal night sky brightness at 00.00 UTC at the stations (from top to bottom) of Labrada, Illas Cíes, Guísamo and Santiago de Compostela. For the sake of clarity the plots, from the lowest on, have been successively shifted by 7 mag_SQM_ arcsec^−2^ along the vertical axis.

**Figure 8.**
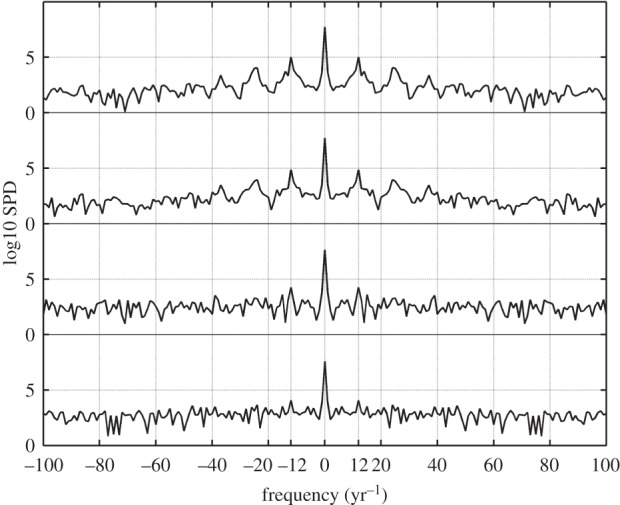
Spectral power density of the yearly time course of the zenithal night sky brightness recorded at 00.00 UTC at the stations (from top to bottom) of Labrada, Illas Cíes, Guísamo and Santiago de Compostela.

The progressive loss of the moonlight modulation as the observing site approaches an urban centre can be assessed from figure 7, which displays the time evolution of the brightness readings taken at 00.00 UTC (middle row of the distributions shown in figure 6) during all consecutive days of the year. The upper plot corresponds to the rural Labrada site and the bottom one to the urban centre of Santiago de Compostela. The severity of this loss can be quantified by analysing the Fourier spectra of these time signals. Their spectral power density (SPD), i.e. the squared modulus of their Fourier transform, is displayed in figure 8. As a measure of this loss, we may define the moonlight modulation factor *γ *= *Φ*(*v*_1_)/*Φ*(0), where *Φ*(*v*_1_) is the SPD of the fundamental harmonic of the NSB signal (located at the frequency *v*_1_≈12 yr^−1^), normalized by the value of the SPD at the origin, *Φ*(0). The values of this index for the different stations of the network are shown in the last column of table 1. These values fall in the range 0.2–0.3 × 10^−3^ for urban sites, 0.3–0.4 × 10^−3^ for periurban areas, 1.2–1.5 × 10^−3^ for transition regions and 1.5–2.4 × 10^−3^ for dark rural sites. The anomalously high value found at the Illa de Sálvora station (2.81 × 10^−3^) is partly an artefact due to the short temporal length of the data segment available for this station (four months), that significantly reduced the size of the window used to calculate the spectrum.

## 4. Discussion

The results presented in this work are affected by several limitations. On the one hand, resorting to the use of low-cost light sensors comes at a price, namely, the photometric band of the SQM-LR detectors is not fully coincident with the standard Johnson–Cousins V band. This fact does not hinder the accuracy and significance of the measurements gathered by the network, but it makes it necessary to perform some spectrum-dependent numerical transformations between photometric systems before these measurements can be directly compared with the evaluations of the NSB made with traditional astrophysical equipment. On the other hand, the size and spatial distribution of this first phase of the network has been limited by financial constraints and some technical requirements: it would be highly desirable to locate measurement stations in all the main urban nuclei of the zone, in order to be able to track with enough accuracy the long-term tendencies of the overall light emissions from the most intense light polluting cities.

Our results are in overall agreement with previous reports on the behaviour of the artificial NSB in 44 selected sites around the globe [41]. The disruption of the natural night sky darkness, the different time course of the brightness in urban and rural setings due to the progressive switch-off of static light sources (ornamental, commercial and domestic) and the reduction of the road traffic flow across the night, the multiplicative or blocking effects of the clouds, the increased variability under cloudy skies, or the reduced amplitude of the moonlight modulation signal in urban and periurban areas are nowadays present worldwide. Any quantitative comparison between studies must take into account, however, the different metrics used to describe the typical sky brightness under different illumination or atmospheric conditions. For instance, the cloud amplification factor for urban and periurban sites, dealt with in §3.2 above, is estimated from the brightness of the peaks (modes) associated with clear and cloudy nights in bimodal histograms like the ones displayed in the top row of figure 3, whereas in [41] the corresponding ‘brightening factor’ is defined in terms of the 28th and the 81st percentile of the sky radiance observations when no data on the fractional cloud cover were available, and as the median radiance observed within ±15 min of midnight (defined as the hour that falls closest to the time when the Sun reaches its deepest point below the horizon for each observing site), if the cloud cover fraction was known from direct meteorological observations. The brightening factors reported in [41] for urban and suburban sites span the range 1.5–17.6, with 70% of values concentrated in the segment 9.2–17.6, whereas most of our values are in the range 10.0–16.0, with a single record equal to 25.

The amplification factors reported here are also consistent with the theoretical predictions made by Aubé *et al.* [42] for the visible range of the spectrum. According to standard radiative transfer models, the spectral radiance at the observer location depends on several variables, among them the spatial distribution and angular radiation pattern of the light sources, the distribution of scattering centres in the atmosphere (molecular and aerosol concentration profiles), and the chemical composition, size and shape of the aerosol particles. The radiance amplification due to the clouds depends additionally on their type, optical properties (optical density, albedo), structure and size, and on the altitude of the cloud base. According to Aubé *et al*., the predicted (and observed) cloud amplification factors are larger for longer wavelengths, and tend to achieve maximum values for low-altitude clouds (0.4–1.0 km). Typical amplification factors do not exceed values of about 20 for mid-altitude clouds, although considerably higher factors are theoretically possible and values as large as 30 have been experimentally reported [42]. Whereas the broad spectral band of the SQM detectors does not allow us to carry out a spectrally resolved analysis, our results support the expected orders of magnitude provided by the theoretical predictions. The relatively high cloud amplification factor recorded at the station of Santiago de Compostela, located at 305 m.a.s.l., is consistent with these predictions, taking into account that this city has frequent nights with skies overcast by low-altitude clouds.

The data analysed in this work correspond to the period 1 January to 31 December 2015. The whole dataset has been used for the analysis, without attempting any detailed study of the seasonal variability. A different behaviour of the statistical parameters of the NSB throughout the seasons of the year is, however, expected: natural celestial sources, among them the Milky Way, are located at different positions in the sky depending on the local time and the date of the year, and they will contribute in a variable amout to the recorded measurements. The most important source of inter-seasonal variability will probably be related to the changing meteorological conditions. Additional variability at local scale can also be anticipated, driven, among other, by sociological factors, e.g. the seasonal increase of population and light use in coastal municipalities during the summer period.

Several relevant studies remain to be done, and will be the subject of future work. One of them is building models from first principles to determine the expected statistical distribution of the zenithal NSB values. These models should provide a sound theoretical basis for the definition of numerical indices to characterize the light pollution status, from the standpoint of the artificial skyglow, of any individual site. They are expected to generalize and improve the significant magnitude index (*m*_1/3_) and the moonlight modulation factor (*γ*) proposed in this work. Better procedures for filtering individual data acquired under particular conditions, among them snow cover and lightning, are also advisable. Providing the network with some spectral resolution capacity is a must, if changes in the spectral composition of the scattered artificial light are to be monitored. The spectral blue-shift associated with the progressive substitution of solid-state light sources (light-emitting diodes) for conventional gas-discharge lamps (high-pressure sodium and metal halides) makes it advisable to develop these systems in the short term.

## 5. Conclusion

As shown by Falchi *et al.* [4], the loss of night darkness, an unwanted side-effect of the extension of artificial outdoor lighting systems, is progressively affecting wider areas of the world. Monitoring the artificial light emissions and measuring their effects at the global and local scales is a necessary step for a better understanding of this phenomenon, and a prerequisite for making informed decisions on public policies concerning outdoor lighting systems.

The detector network described in this paper enables the continuous monitoring of one of the most conspicuous manifestations of these effects, the anthropogenic zenithal NSB, at a countrywide level. Its full integration within a public meteorological and environmental information service provides some distinct advantages and economies of scale. The NSB data complement the daytime irradiance measurements usual in all meteorological networks, allowing in this way a continuous assessment throughout the day of the levels of optical radiation at the observation sites. On the other hand, integrating a network of low-cost sensors in an already existing meteorological infraestructure is a cost-effective way of handling the need of acquiring, transmitting and storing a high volume of environmental data in continuous operation mode, and significantly facilitates its public dissemination.

The data gathered by this network reveal the significant increment of the zenithal NSB over its naturally expected values, as well as the strong amplification effect due to the light diffusely reflected by the clouds in urban and periurban sites. These data also show the loss of amplitude of the night-time light modulation due to the Moon, as we approach the urban nuclei. Two quantitative indices, the significant magnitude (*m*_1/3_) and the moonlight modulation factor (*γ*), are defined and used in this work to characterize the strength of these effects.

## Data accessibility

Ten-minute averages of the NSB readings are displayed in (and can be downloaded from) the MeteoGalicia website [34]. Data streams of the last few days can also be seen in this page as graphic plots. Extended period datasets can be downloaded in different file formats together with the meteorological variables of interest.

The native 1 min sampling rate datasets used in this study are freely available upon request to salva.bara@usc.es or directly downloadable from Dryad http://dx.doi.org/10.5061/dryad.kv072 [43].
